# Discontinuation of *Pneumocystis jirovecii* Pneumonia Prophylaxis with CD4 Count <200 Cells/µL and Virologic Suppression: A Systematic Review

**DOI:** 10.1371/journal.pone.0028570

**Published:** 2011-12-16

**Authors:** Cecilia T. Costiniuk, Dean A. Fergusson, Steve Doucette, Jonathan B. Angel

**Affiliations:** 1 Division of Infectious Diseases, Ottawa Hospital, Ottawa, Canada; 2 Ottawa Hospital Research Institute, Ottawa, Canada; University of California Los Angeles, United States of America

## Abstract

**Background:**

HIV viral load (VL) is currently not part of the criteria for *Pneumocystis jirovecii* pneumonia (PCP) prophylaxis discontinuation, but suppression of plasma viremia with antiretroviral therapy may allow for discontinuation of PCP prophylaxis even with CD4 count <200 cells/µL.

**Methods:**

A systematic review was performed to determine the incidence of PCP in HIV-infected individuals with CD4 count <200 cells/µL and fully suppressed VL on antiretroviral therapy but not receiving PCP prophylaxis.

**Results:**

Four articles examined individuals who discontinued PCP prophylaxis with CD4 count <200 cells/µL in the context of fully suppressed VL on antiretroviral therapy. The overall incidence of PCP was 0.48 cases per 100 person-years (PY) (95% confidence interval (CI) (0.06–0.89). This was lower than the incidence of PCP in untreated HIV infection (5.30 cases/100 PY, 95% CI 4.1–6.8) and lower than the incidence in persons with CD4 count <200 cells/µL, before the availability of highly active antiretroviral therapy (HAART), who continued prophylaxis (4.85/100 PY, 95% CI 0.92–8.78). In one study in which individuals were stratified according to CD4 count <200 cells/µL, there was a greater risk of PCP with CD4 count ≤100 cells/µL compared to 101–200 cells/µL.

**Conclusion:**

Primary PCP prophylaxis may be safely discontinued in HIV-infected individuals with CD4 count between 101–200 cells/µL provided the VL is fully suppressed on antiretroviral therapy. However, there are inadequate data available to make this recommendation when the CD4 count is ≤100 cells/µL. A revision of guidelines on primary PCP prophylaxis to include consideration of the VL is merited.

## Introduction

Currently, The National Institutes of Health, the Centers for Disease Control and Prevention and the HIV (Human Immunodeficiency Virus) Medicine Association of the Infectious Diseases Society of America guidelines state that prophylaxis against *Pneumocystis jirovecii* pneumonia (PCP) may be discontinued safely in HIV-infected individuals when CD4 counts are maintained >200 cells/µL for over three months on antiretroviral therapy [1]. This recommendation was made when prospective observational and randomized controlled trials in the highly active antiretroviral therapy (HAART) era demonstrated that discontinuation of PCP prophylaxis was safe when a sustained CD4 count >200 cells/µL was achieved [2]–[4].

Medications used for PCP prophylaxis include trimethoprim/sulfamethoxazole (TMP-SMX), pentamidine, dapsone and atovaquone. Adverse effects include anemia, hyperkalemia, renal dysfunction, hypersensitivity reactions, rashes and hepatitis and may occasionally be life-threatening [5], [6]. TMP-SMX, the first-line agent for PCP prophylaxis, may be associated with hypersensitivity reactions such as Stevens-Johnson Syndrome [5], [6] and have been reported in up to 4% of the HIV population [6]. Other disadvantages of prophylactic medications include additional pill burden, drug interactions, and cost. For individuals on inhaled pentamidine, there is also the inconvenience of monthly clinic visits [7].

HIV viral load (VL) and changes in VL have clearly been shown to be associated with the risk of development of OIs, independent of CD4 count [7], [8]–[11]. Mellors et al. examined longitudinal data of 1,640 HIV patients registered in the Multicenter AIDS Cohort Study (MACS). They demonstrated that median HIV-1 RNA explained 51% and 58% of the variability in AIDS and death, and median CD4 count accounted for 29% and 35% of this variability, respectively. The authors concluded that, in patients with untreated HIV infection, a single HIV-1 RNA measurement was the strongest predictor of time to AIDS and death, explaining about half of the variability in these clinical outcomes [10]. However, at present the HIV-RNA level, or VL, is not part of the criteria used to guide recommendations for discontinuation of PCP prophylaxis, which are based almost entirely on CD4 counts [7].

Our goal was to perform a systematic review of the published studies to determine the incidence of PCP in HIV-infected individuals with fully-suppressed plasma VL on antiretroviral therapy who discontinued PCP prophylaxis with CD4 count <200 cells/µL. We then aimed to compare this incidence to 1) the natural history rate of PCP in the HIV-infected population, 2) the incidence of PCP in individuals who discontinued prophylaxis with CD4l count >200 cells/µL and 3) the incidence of PCP in individuals who continue prophylaxis with CD4 count <200 cells/µL prior to the availability of HAART. If it could be demonstrated from this evidence that individuals whose CD4 count is <200 cells/µL and VL are suppressed on antiretroviral therapy could discontinue prophylaxis with a risk of acquiring PCP similar to individuals in the other three groups, this finding would have important clinical implications and would indicate that a revision of clinical guidelines may be merited.

## Methods

### Literature Search

We conducted a systematic search of the literature to retrieve articles examining the incidence of PCP in HIV-infected individuals with fully-suppressed plasma VL on antiretroviral therapy who discontinued PCP prophylaxis with CD4 count <200 cells/µL, We searched OVID Medline (1950-August 8 2010), EMBASE (1947 to 2010 week 31), the Cochrane Central Registry of Controlled Trials (2005 to September 2010) and the Cochrane Database of Systematic Reviews (3^rd^ quarter 2010) for original studies and systematic reviews. Search terms used included “pneumonia,” “pneumocystis,” “pneumocystosis or carinii,” “antibiotic prophylaxis or chemoprevention or preventative or prevention,” “HIV infection or human immunodeficiency virus or AIDS or acquired immune deficiency syndrome,” and “discontinue or cease or stop or cessation or halt or terminate or suspend.” Titles and abstracts were reviewed independently by two investigators. When there was uncertainty regarding the suitability of a study, full text articles were retrieved and the articles were discussed between the two investigators until agreement was reached. Bibliographies of several studies were also reviewed to ensure all relevant studies were captured.

Studies examining 1) the natural history rate of PCP in the HIV-infected population, 2) the incidence of PCP in individuals who discontinued prophylaxis with CD4 count >200 cells/µL and 3) the incidence of PCP in individuals who continue prophylaxis with CD4 count <200 cells/µL prior to the availability of HAART were retrieved by examining the reference list of the Guidelines for prevention and treatment of opportunistic infections in HIV-infected adults and adolescents: recommendations from CDC, the National Institutes of Health, and the HIV Medicine Association of the Infectious Diseases Society of America [1].

### Eligibility

Studies in the English language were eligible. Abstracts were not included.

### Study Selection and Data Extraction

To address our primary study question, studies were selected if they examined the incidence of PCP in adults who met the following inclusion criteria: 1) HIV-infected, 2) CD4 count <200 cells/µL, 3) fully suppressed VL as measured by conventional assays 4) on antiretroviral therapy, and 5) discontinuation of PCP prophylaxis (primary or secondary) or not already taking prophylaxis. Studies in the other three categories were selected if they were referenced in the current guidelines for prevention and treatment of opportunistic infections in HIV-infected adults and adolescents [1], recognizing that other studies in these categories have been published. Data were extracted by two reviewers.

### Analysis

Incidence rates of PCP in persons who discontinued prophylaxis with CD4 count <200 cells/µL and fully suppressed VL on antiretroviral therapy were compared to 1) the natural history rate of PCP in the HIV-infected population, 2) the incidence of PCP in individuals who discontinued prophylaxis with CD4 count >200 cells/µL on antiretroviral therapy and 3) the incidence of PCP in individuals who continued prophylaxis with CD4 count <200 cells/µL prior to the availability of HAART.

## Results

**Figure 1:**
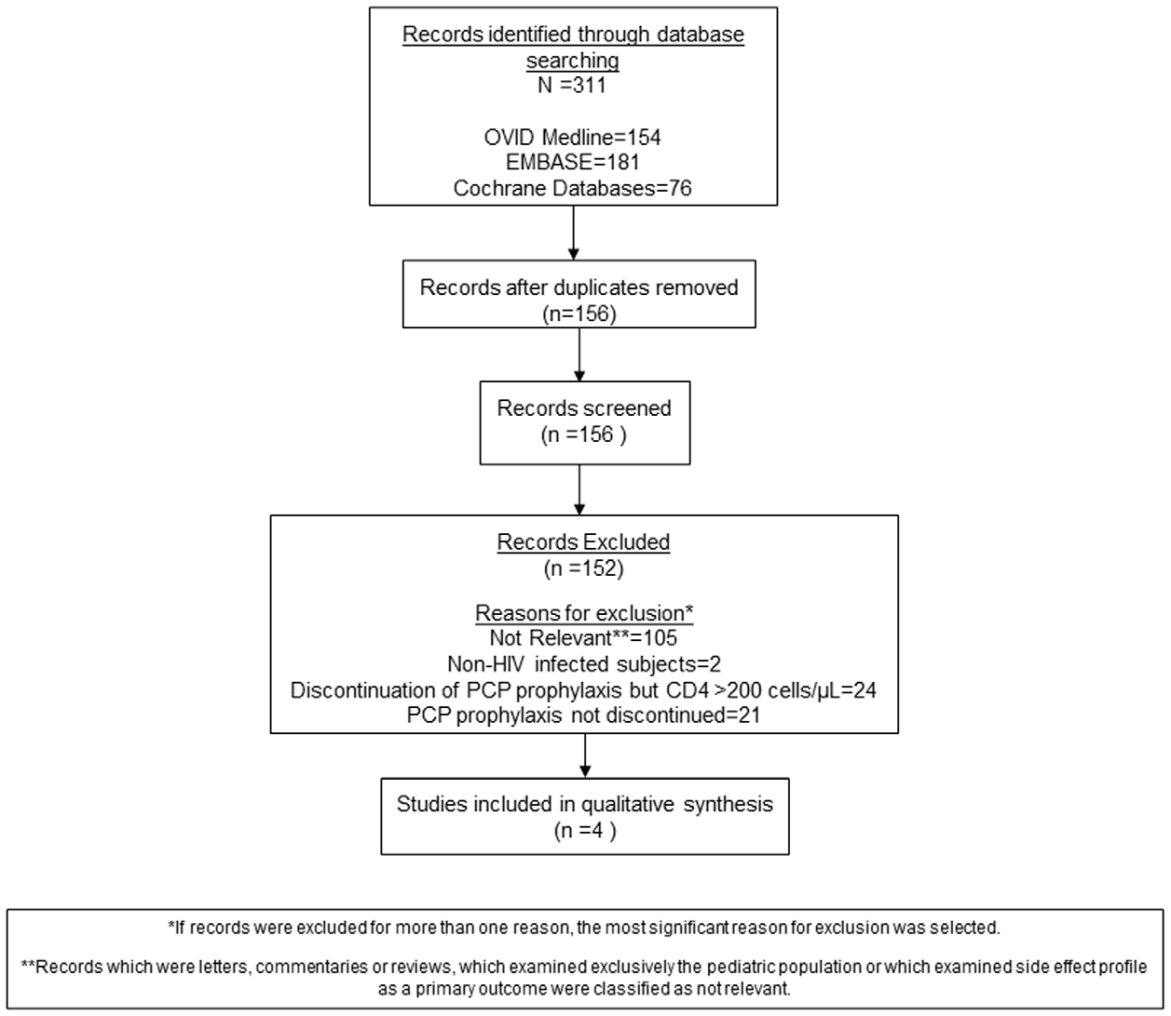
Flow diagram of literature search for systematic review.

OVID Medline search yielded 154 results, EMBASE yielded 181 results and the Cochrane Databases yielded 76 results. Four studies contained data which addressed our study question (Figure 1). Table 1 includes data on clinical characteristics and Table 2 includes methodological characteristics of the studies. Table 3 provides incidence rates of PCP.

**Table 1:** Clinical Characterization of Studies on Discontinuation of *Pneumocystis jirovecii* Pneumonia (PCP) Prophylaxis.

Author	Inclusion Criteria	VL for inclusion (copies/mL)	1° proph (Yes/No)	2° proph (Yes/No)	Sex (M/F)	Age (Years)	CD4 at time of prophylaxis discontinuation (mean cells/µL)	CD4 at end of study (mean cells/µL)	VL at end of study (mean copies/mL)
**Soriano V et al ** [**4**]	CD4 count >100 cells/µL and VL <500 copies/mL after 3 months of initiating HAART	<500	Y	Y	NR	NR	NR	NR	NR
**D'Egidio G et al ** [**12**]	CD4 count stable <200 cells/µL and on effective HAART as indicated by VL <50 copies/mL for at least 3 months	<50	Y	Y	15/4	47 median	118	136	<50
**COHERE study group ** [**14**]	Persons from cohorts with prospectively registered start and stop dates of specific therapeutic and prophylactic regimens against PCP with follow-up time after January 1998 and started on HAART on or after this date; ≥1 CD4 count and VL obtained during the follow-up period	<400	Y	N	NR	NR	NR	NR	NR
**Cheng CY et al ** [**15**]	Baseline CD4 count <200 cells/µL and more than 3 months on HAART	<400	Y	Y	NR	NR	NR	NR	NR

NR = not reported for subpopulation of interest.

**Table 2:** Methodological Characterization of Studies on Discontinuation of PCP prophylaxis.

Author	Recruitment Period	Country	Study Designs	Single vs Multiple Centres	Primary Outcomes	Outcome Definitions
**Soriano V et al ** [**4**]	March 1997–March 1998	Spain	Case series	Single	Opportunistic Infections	NR
**D'Egidio G et al ** [**12**]	NR	Canada	Prospective cohort	Single	PCP	CDC
**COHERE study group ** [**14**]	1997 until NR	29 European Countries	Prospective cohort (12 cohorts)	Multiple	PCP	CDC
**Cheng CY et al ** [**15**]	April 1997–September 2007	Taiwan	Prospective observational	Single	PCP	CDC

NR = nor reported for subpopulation of interest.

CDC = Centre for Disease Control Definition of PCP.

**Table 3:** Incidence Rates of PCP with Discontinuation of PCP prophylaxis with CD4 count ≤200 cells/µL and Fully Suppressed Viral Load (VL).

Author	Duration of follow-up (Patient Years)	N analyzed	Events	Rate/100 PY (95% Confidence Interval)
**Soriano V et al ** [**4**]	42	28	0	0 (0, 8.78)
**D'Egidio G et al ** [**12**]	21.75	19	0	0 (0, 17.0)
**COHERE study group ** [**14**]	674.6*	NR*	4*	0.59 (0.16, 1.52)*
CD4≤100 cells/µL	104.2	NR	4	3.84 (1.05, 9.83)
CD4 100–200 cells/µL	570.4	NR	0	0 (0, 0.65)
**Cheng CY et al ** [**15**]	323	215	1	0.31 (0.01, 1.71)
**Pooled**				**0.48 (0.06, 0.89)**

NR = not reported for subpopulation of interest.

*Based on combined data for CD4≤100 and 101–200 cells.

Studies differed with respect to study design, populations studied, sample size, follow-up duration and methods of data analysis. The first study was a case series in which Soriano et al. [4] retrospectively reviewed outcomes at 18 months in 53 patients from Spain in whom secondary prophylaxis for various opportunistic infections was discontinued. Twenty-nine of these patients had been receiving secondary prophylaxis for PCP. Prophylaxis was discontinued if both the CD4 count was >100 cells/µL and VL was <500 copies/mL after three months of HAART. Mean (range) baseline CD4 count prior to initiating antiretroviral therapy was 114 (3–188) cells/µL and VL was 28,456 (1834–345,879) copies/mL. The CD4 counts at which patients discontinued secondary prophylaxis were not reported. At the time of the study, conventional VL load assays could only measure HIV-RNA as low as 500 copies/mL. Of the 53 patients, only one person developed PCP. However, the patient had interrupted her antiretrovirals for 6 weeks due to gastrointestinal upset, and consequently had a VL>500, 000 copies/mL and CD4 of 46 cells/µL [4]. Thus, if this patient were excluded, the incidence of PCP would be zero cases over 42 person-years follow-up (PYFU).

The second study was a prospective cohort study of 19 patients in which D'Egidio et al. [12] followed patients from Ottawa, Canada with CD4 counts having plateaued at <200 cells/µL and undetectable VL while off PCP prophylaxis. During this study in 2007, VL assays which detect virus down to 50 copies/mL, were in use. There were no episodes of PCP during 261 patient-months of follow-up [12]. The authors compared the incidence of PCP to the incidence in 1665 patients at the Multicenter AIDS Cohort Study (MACS) [13] who were not on HAART and not receiving any prophylaxis against PCP with CD4 counts <200 cells/µL. As approximately 20 cases of PCP per 100 patient-months of HAART would have been expected, d'Egidio et al. determined that the risk of developing PCP while on HAART with a CD4 count <200 cells/µL was significantly different from the risk in untreated HIV (rate difference 9.2%; 95% CI 5.7, 12.8%, p<0.05) [12], [13].

The third study was performed by The Opportunistic Infections Project Team of the Collaboration of Observational HIV Epidemiological Research in Europe (COHERE), a collaboration of 33 cohorts in 29 European countries which examines prognosis and outcome in HIV-infected individuals from across Europe. These cohorts include approximately 240,000 adults, 6,400 children and 28,000 mother-infant pairs. The COHERE study [14] was a prospective study of 12 cohorts in Europe which analyzed the incidence and risk factors for primary PCP. The incidences of primary PCP were calculated after stratification by current use of PCP prophylaxis (“on” or “off”) and VL. Another analysis was performed whereby the incidence of primary PCP after discontinuing prophylaxis in patients having initiated HAART was performed. This latter analysis was relevant to our systematic review. In this latter analysis, patients who had never initiated primary PCP prophylaxis or discontinued prior to starting HAART were excluded. In the COHERE study patients were also stratified according to CD4 count, and thus is the only study providing data about the incidence of PCP in persons with CD4 counts ≤100 cells/µL and suppressed VL discontinuing prophylaxis. Two data sets were of interest to us (CD4 ≤100 and VL <400, and CD4 101–200 and VL <400, who discontinued prophylaxis). For individuals with CD4 counts between 101–200 cells/µL and VL <400 copies/mL, there were no episodes of PCP over 570.4 PYFU, thus yielding an event rate of 0 events per 100 PYFU (95% CI, 0.0, 0.65) [14]. In patients with CD4 counts ≤100 cells/µL and VL <400 copies/mL, there were 4 episodes of PCP over 104.2 person-years follow-up PYFU, for an event rate of 3.84 events per 100 PYFU (95% CI, 1.05, 9.83) [14].

In the fourth and last study, which originated from Taiwan, Cheng et al. [15] examined the incidence of PCP in three groups of subjects who discontinued primary and secondary prophylaxis and those who had never initiated prophylaxis for PCP according to guidelines. The subpopulation of interest for our review was the 215 patients who achieved undetectable VL and had early discontinuation of primary (n = 130) or secondary (n = 85) prophylaxis for PCP. A single patient who had been receiving secondary prophylaxis developed PCP after a total period of observation of 323 PY, giving an incidence rate of 0.31 episodes/100 PY (95% CI, 0.01, 1.72). This was similar to the incidence in patients who continued prophylaxis until their CD4 count was >200 cells/µL (incidence rate 0.45 cases/100 PY (95% CI 0.05, 1.63) and adjusted risk ratio 0.63; 95% CI 0.03, 14.89) [15].

**Figure 2:**
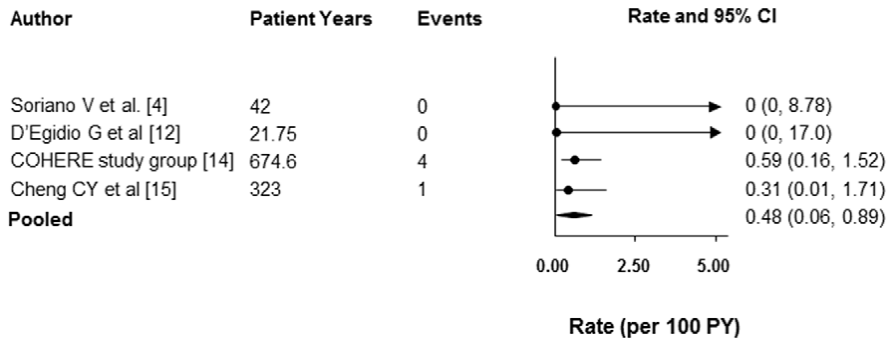
Incidence of PCP in HIV-infected Individuals on Antiretroviral Therapy who Discontinue Prophylaxis with CD4+ Count <200 cells/µL with Suppressed Viral Load.

Figure 2 demonstrates the incidence rates (IR) for PCP in patients with CD4 counts <200 cells/µL and suppressed VL who discontinued prophylaxis. The overall incidence of PCP when these studies are combined is 0.48 events/100 PY (95% CI 0.06, 0.89). As only the COHERE study stratified patients based on CD4 count ≤100 cells/µL and 101–200 cells µL, we were unable to stratify the overall incidences of PCP based on CD4 count. Furthermore, only the COHERE study examined incidence of PCP in the context of discontinuation of primary prophylaxis. Although the other three studies examined patients discontinuing both primary and secondary prophylaxis, only Cheng et al. reported data separately for the discontinuation of primary and secondary prophylaxis. Hence, we were unable to report incidence rates of PCP for discontinuation of primary versus secondary prophylaxis in this systematic review. Furthermore, as the COHERE study did not indicate the number of individuals with CD4 counts <200cells/µL and suppressed VL on HAART who were adults, it was not possible for us to include rates from the COHERE study in our systematic review which necessarily applied to adults only.

**Figure 3:**
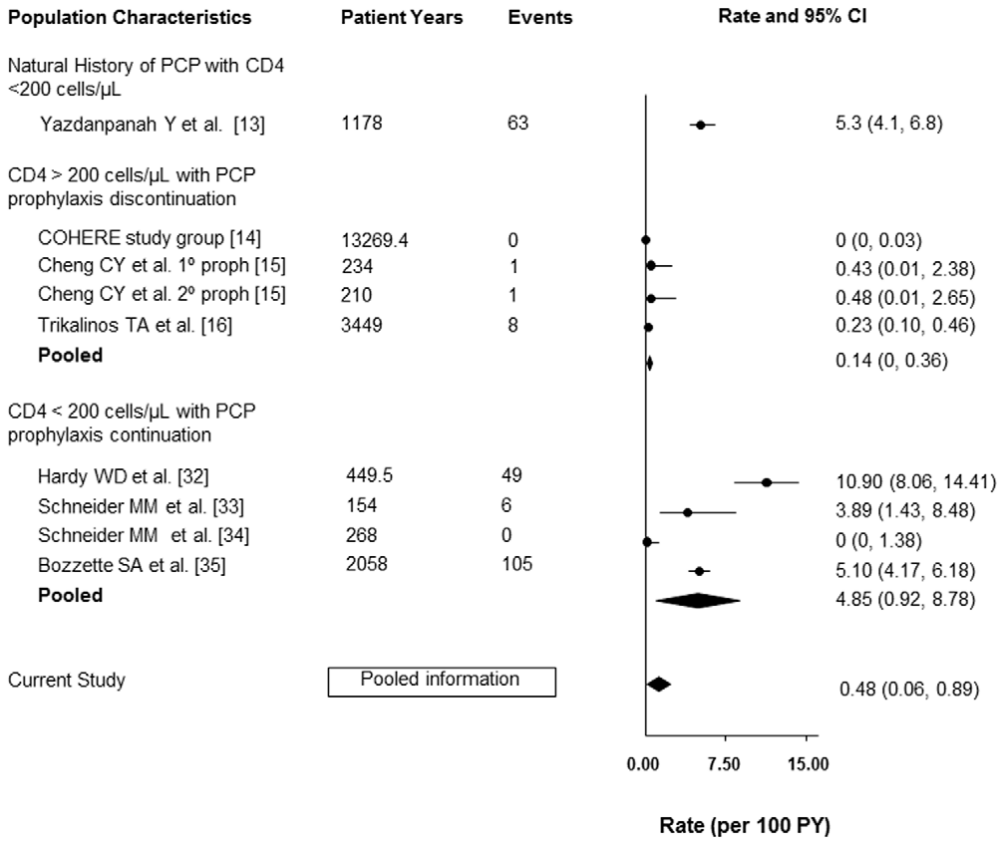
Incidence of PCP in HIV-infected Individuals in Various Situations.

Figure 3 depicts this incidence compared to the incidence of PCP in untreated HIV infection (5.30 events/100 PY, 95% CI 4.1, 6.8). This incidence rate is the pooled rate based on the incidence of PCP at different CD4 strata in two cohorts reported by Yazdanpanah et al. (3.1 cases/100 PY, 95% CI 2.0, 4.6 for individuals with CD4 count 101–200 cells/µL, 6.7 cases/100 PY (95% CI 3.7, 11.7) for individuals with CD4 51–100 cells/µL and 11.4 cases/100 PY for individuals with CD4 count 0–50 cells/µL (95% CI 7.5, 17.3) [13]. Figure 3 also depicts the incidence of PCP in patients with CD4 counts >200 cells/µL with discontinuation of prophylaxis (0.14 events/100 PY, 95% CI 0, 0.36) [14]–[16]. The result was based on data from the COHERE and Cheng studies, as well as a meta-analysis by Trikalinos et al. [16]. The meta-analysis by Trikalinos et al. included 14 studies from 1998 until 2001 inclusive [2], [3], [17]–[28]. Results from the COHERE, Cheng et al. and Trikalinos et al. studies were included as they capture the majority of studies examining the incidence of PCP in patients with CD4 counts >200 cells/uL who discontinue prophylaxis. Data that were published after the meta-analysis by Trikalinos et al. include studies by Abgrall et al [29], Mussini et al [30] and Zellweger et al [31]. However, incorporation of data from these later studies did not have an impact on the reported rate of PCP (range 0.00–1.2 per 100 person-years).

The incidence of PCP in patients with CD4 counts <200 cells/µL, before the availability of highly active antiretroviral therapy and who were receiving PCP prophylaxis, is also demonstrated in Figure 3 (4.85events/100 PY, 95% CI 0.92–8.78) [32]–[35]. The values used to calculate the latter event rate were extracted from the studies cited in the current guidelines for the prevention of OIs in HIV-infected adults and adolescents [1], recognizing that there are additional published reports on this topic. The rates of PCP in the aforementioned categories are well-established and have been used frequently in the development of various treatment guidelines.

## Discussion

Recommendations for chemoprophylaxis discontinuation are based on CD4 counts [1]. However, there are limitations with using the peripheral blood CD4 count as the sole marker of immune response. It is estimated that only 2% of the total pool of CD4 T cells are present in blood at any time, and that even a small shift in the distribution of cells between the lymphoid tissues and blood cells may result in a large alteration in peripheral blood CD4 count [36]. In many studies suggesting it is safe to discontinue PCP prophylaxis when CD4 count is >200 cells/µL, this rise in CD4 count was accompanied by a decline in VL [7]. Furthermore, patients with similar CD4 counts frequently progress at different rates despite taking the same medications [8]. These findings suggest that CD4 count alone is not the sole predictor of susceptibility to PCP.

Improvement in immune function associated with lower VL has been observed when assessing responses to vaccinations. For example, lower VLs have been associated with improved responses to hepatitis A, hepatitis B and varicella vaccinations independent of CD4 count [37]–[39]. In a large survey of an HIV-infected cohort in the United States, a protective effect of PPV-23 vaccination was also shown against all-cause pneumonia, and this benefit was lost in patients with VL >100,000 copies/mL, irrespective of the CD4 count [40]. Moreover, in a randomized, double-blind comparative trial of subunit and virosomal influenza vaccines for immunocompromised patients, suppression of VL with HAART was a more important predictor of response to influenza vaccination than the CD4 count [41].

In addition to discontinuation of prophylaxis after initiation of HAART, the COHERE study also analyzed the general incidence of PCP in persons who either never initiated prophylaxis or discontinued prophylaxis prior to initiating HAAART. They demonstrated an overall event rate of 0.12 events per 100 PYFU (95% CI 0.02–0.45) when the CD4 count is between 101–200 cells/µL and VL is suppressed in patients not receiving prophylaxis. This was similar to the event rate of 0.21 episodes per 100 PYFU (95% CI 0.08–0.43) in persons with CD4 counts between 101–200 cells/µL and suppressed VL receiving prophylaxis. The low overall incidence rate of PCP when CD4 count is 101–200 cells/µL may serve as supporting evidence that prophylaxis may not be required in patients with suppressed VL and CD4 count in the 101–200 cells/µL range.

Another important observation from the COHERE study was that the risk of PCP differed based on the CD4 strata in individuals who discontinued prophylaxis after initiating HAART. While it appeared safe to discontinue prophylaxis with CD4 count between 101–200 cells/µL, the upper confidence interval level of the incidence rate in persons who discontinued prophylaxis with CD4 count ≤100 cells/µL after initiating HAART was high (9.83 events per 100 PYFU), suggesting an unacceptably high risk of PCP in this one study.

The studies included in this review differed in several important aspects. Most notably, not all studies examined the same primary outcome (i.e. incidence of PCP during follow-up in patients off prophylaxis with CD4 counts <200 cells/µL and suppressed VL), thus we were unable to perform a meta-analysis. Furthermore, some studies examined discontinuation of PCP primary prophylaxis whereas others examined discontinuation of PCP secondary prophylaxis. Individuals receiving PCP prophylaxis for primary compared to secondary prophylaxis may differ in that having had PCP in the past increases one's risk for the development of subsequent episodes [1], [9]. Moreover, in addition to adults, the COHERE cohorts also include children and mother-infant pairs. However, the number of adults, children and mother-infant pairs who had CD4+ <200 cells/µL and suppressed VL on HAART is not reported.

Studies also differed significantly with respect to sample sizes and follow-up conditions. Sample size ranged from 19 subjects in the D'Egidio study to greater than 20, 000 in the COHERE study. In the COHERE study, one individual could provide several episodes of PCP to one CD4/VL stratum, while in the other studies individual follow-ups are examined. In the Soriano et al. study, follow-up included time with CD4 count >200 cells/µL, therefore likely underestimating the incidence of PCP and overestimating safety by prolonging follow-up. In the Cheng et al. study, follow-up time included persons with detectable VLs, thus likely overestimating the incidence. Follow-up durations whereby patients were off prophylaxis with CD4 counts <200 cells/µL and suppressed viremia differed among studies. Finally, studies were performed at different time points and current VL assays have increased sensitivity compared to those used decades ago. Whereby older assays could only measure VL to 400–500 copies/mL, newer assays can measure VL down to 40–50 copies/mL. This may be important due to deleterious effects associated with even low level plasma viremia, such as direct viral cytopathogenecity, persistent immune activation with apoptosis of uninfected cells, and decreased thymic output of naïve CD4 T cells [37].

Limiting the use of prophylaxis against PCP to situations where it has been shown to be most beneficial will minimize adverse drug reactions. The AIDS Clinical Trials Group (ACTG) 081 was a randomized clinical trial (RCT) of 842 HIV-infected individuals which compared the effectiveness of three alternative prophylactic regimens. There was no difference among treatment arms with regards to adverse events. Prevalence of patient self-reported fatigue was 67%, headache 61%, sleep disturbance 50% and dermatological problems 46%. Fever, sense of imbalance, paresthesias, diarrhea and cough all had self-reported prevalences greater than 30%, demonstrating a substantial burden of adverse effects [42]. In an earlier meta-analysis by Ioannidis et al., which examined RCTs comparing efficacy and toxicity of different PCP prophylaxis regimens, trimethoprim-sulfamethoxazole was almost universally efficacious at preventing PCP when tolerated. The risk of discontinuing trimethoprim-sulfamethoxazole due to adverse reactions depended on dosing schedule and decreased by 43% if one double-strength tablet was given three times weekly instead of daily. For dapsone, among 100 patients given 100 mg orally once daily instead of twice weekly over a year, the authors determined that 7 fewer patients would develop PCP but 17 more would have significant toxic reactions [43]. Of the 4 studies included in our systematic review, only Cheng et al. reported the incidence adverse effects due to PCP prophylaxis. They estimated the crude incidence of adverse effects for primary prophylaxis with trimethoprim-sulfamethoxazole to be 23.3%, of which 13.3% were due to skin rashes, 6.8% due to leukopenia, 2.9% due to gastrointestinal intolerance and 0.4% due to Stevens-Johnson syndrome. Of 47 patients (21.8%) who developed adverse events leading to discontinuation of secondary prophylaxis, adverse events included skin rashes in 17.6%, gastrointestinal intolerance in 2.3% and leukopenia in 1.9% [15]. However, it should be noted that these rates do not apply exclusively to individuals with CD4 counts <200 cells/µL with suppressed VL on HAART.

An important limitation of our systematic review is the heterogeneity between the studies examined. Although this precluded us from performing a meta-analysis, we pooled results to include the totality of available, relevant evidence in our systematic review. Another limitation of our study is its retrospective nature in which we were dependent upon the availability and accuracy of data reported by others. Furthermore, none of the studies included were randomized controlled trials, eliminating the ability to control for confounding factors which may have influenced results.

The question of whether it is safe to discontinue prophylaxis with suppressed VL and CD4 count <200 cells/µL would be best addressed with an RCT. However, this is unlike unlikely to occur in the foreseeable future. As a meta-analysis was not possible due to the heterogeneity between studies, here we conducted a systematic review of the available evidence. Though studies are few in number, when examined together there is sufficient evidence to demonstrate that the risk of PCP infection in HIV-infected individuals on HAART with suppressed VL is sufficiently low to allow discontinuation of primary prophylaxis, despite a CD4 count between 101–200 cells/µL. Given the low overall risk of PCP with CD4 count between 101–200 cells/µL, combined with the drawbacks of prophylaxis for OIs, we suggest a revision of clinical guidelines for primary prophylaxis is merited to include consideration of the VL. However, as only one study examined the risk of PCP prophylaxis discontinuation at different CD4 strata, including ≤100 cells/µL, it may not be appropriate to apply this recommendation to individuals with CD4 counts of ≤100 cells/µL at this time.
